# One-Stop Clinic Utilization in Plastic Surgery: Our Local Experience and the Results of a UK-Wide National Survey

**DOI:** 10.1155/2015/747961

**Published:** 2015-07-05

**Authors:** Mark Gorman, James Coelho, Sameer Gujral, Alastair McKay

**Affiliations:** ^1^Castle Hill Plastic Surgery Unit, Hull HU16 5JQ, UK; ^2^Department of Plastic Surgery, Royal Devon and Exeter Hospital, UK; ^3^Canniesburn Plastic Surgery Unit, Glasgow, UK

## Abstract

*Introduction*. “See and treat” one-stop clinics (OSCs) are an advocated NHS initiative to modernise care, reducing cancer treatment waiting times. Little studied in plastic surgery, the existing evidence suggests that though they improve care, they are rarely implemented. We present our experience setting up a plastic surgery OSC for minor skin surgery and survey their use across the UK. *Methods*. The OSC was evaluated by 18-week wait target compliance, measures of departmental capacity, and patient satisfaction. Data was obtained from 32 of the 47 UK plastic surgery departments to investigate the prevalence of OSCs for minor skin cancer surgery. *Results*. The OSC improved 18-week waiting times, from a noncompliant mean of 80% to a compliant 95% average. Department capacity increased 15%. 95% of patients were highly satisfied with and preferred the OSC to a conventional service. Only 25% of UK plastic surgery units run OSCs, offering varying reasons for not doing so, 42% having not considered their use. *Conclusions*. OSCs are underutilised within UK plastic surgery, where a significant proportion of units have not even considered their benefit. This is despite associated improvements in waiting times, department capacity, and levels of high patient satisfaction. We offer our considerations and local experience instituting an OSC service.

## 1. Introduction

Dating back to the mid-1990s, plastic surgery units have reported issues dealing with growing number of patient referrals in a timely fashion. A significant burden is the diagnosis and treatment of outpatient skin cancer [1, 2]. An internal 2010 audit at the Castle Hill regional UK Plastic Surgery Department identified such a problem, with a failure to manage patient referrals within agreed government time frames. National health care systems employ different waiting time targets. In the UK, it is a legal patient right to receive care (be it surgery or a decision towards conservative management) within 18 weeks of being referred [3, 4]. When struggling to meet the 18-week wait target (18 ww) one strategy has been to streamline care through “see and treat” one-stop clinics (OSCs) [5]. Within UK plastic surgery, the institution of OSCs has been advocated by the National Institute for Health Care and Excellence and the British Association of Plastic Surgery. It was felt to be key to the Department of Health's modernisation strategies (“action on plastic surgery”) [2, 3, 6]. A thorough Medline search indicates that despite such support and evidence of improved service (e.g., cost savings and patient satisfaction) when instituted, OSCs seem to be rarely implemented within plastic surgery [1, 2, 7]. The reasons for this and actual uptake levels across the UK are however unknown. To address these questions, the aims of this study were to conduct a nationwide survey of plastic surgery OSC utilisation and present our local experience setting up an OSC to manage minor outpatient skin cancer surgery. Alongside the primary outcome measure, 18 ww performance, patient satisfaction was also surveyed.

**Figure 1:**
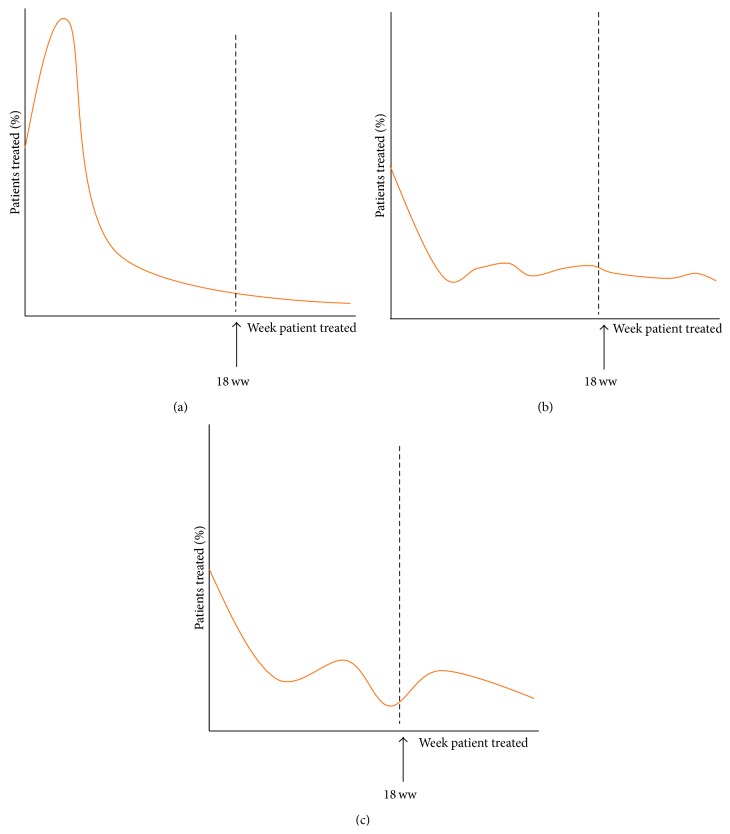
Three illustrative figures reflecting how effectively a department delivers care in relation to the 18 ww pathway. The first graph: (a) depicts an ideal negative “ski slope” shape curve, where the majority of referrals are seen and treated at the beginning of the pathway, implying that a department has adequate resource to cope with demand. This is opposed to a wider distribution in (b) or bimodal pattern in (c), with peaks at either side of the 18 ww threshold (or target) implying an unsuccessful attempt to rush through patients at the end of the pathway in order to meet target compliance (avoiding fines imposed by the NHS) rather than improve patient care, as is intended by the 18 ww.* (a) Ideal “ski slope” curve*.* (b) Wider distribution*,* and (c) bimodal pattern implying a struggle to meet the demand of referrals*.

In relation to cancer, the importance of markers such as the 18 ww relates to evidence spanning the surgical specialities (including head and neck, general, breast, gynaecology, and skin/dermatology) indicating that rapid access to diagnosis and treatment decreases patient anxiety and has been associated with improved survival outcomes [8–13]. Previous studies, when measuring waiting time performance in relation to OSCs, have not done so against nationally agreed benchmarks, and whilst believing this is important, we acknowledge the 18 ww to be just one of the many targets instituted by regulatory bodies to promote shorter waiting times and that such thresholds will inevitably change over time [14]. Based upon work by the Cancer Service Collaborative “Improvement Partnership” (CSCIP 2006), surgical departments in the UK should see and treat ≥ 90% of outpatients (≥95% for inpatients) within the 18 ww [14]. The 10% leeway makes allowance for more complex or unforeseen patient scenarios, such that a service operating in a healthy fashion should have the capacity to see and treat the vast majority of patients in the first few weeks, not spread over the 18 ww pathway [15]. Plotted on a graph, an 18 ww curve should thus be shaped like a ski slope (Figure 1(a)) as opposed to a wide spread or bimodal pattern of distribution (Figures 1(b) and 1(c)) [16, 17]. Such patterns, alongside 18 ww target compliance, may aid the assessment of how a department is functioning (further explanation in Figure 1 caption).

Given that a successful OSC delivers care in one clinical session, that before required two or more, an obvious question is why they are not more routinely employed. The answer within UK plastic surgery is unknown and as mentioned before forms part of our motivation to survey OSCs nationwide. It may be that an absence of “lean thinking” elsewhere, as advocated by the NHS and adopted within our department (when planning and conceiving an OSC), may lead to OSCs not being considered or being incorrectly ruled out [18]. Derived from the automotive industry, “lean thinking” is a management philosophy that aims to improve quality by reducing waste and facilitating flow in care processes [19]. Key to its success in healthcare is an open relationship between department managers and senior clinicians, where it is important that senior clinical staff take a leadership role. All staff, especially frontline workers, should be consulted to help map out “current state” processes and identify waste/rate limiting steps in departmental care pathways [20]. Clear outcome measures must be employed to develop and track interventions (i.e., the 18 ww and validated measures of patient satisfaction) [21]. 

**Figure 2:**
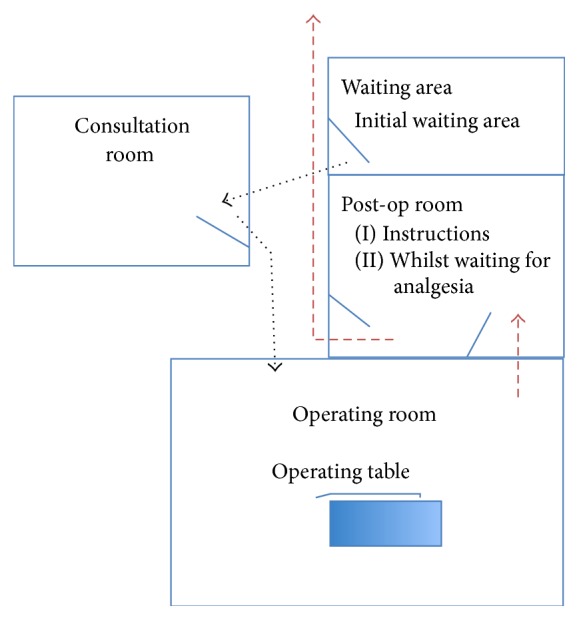
Layout and patient flow and though see the one-stop clinic.

*Organisation and Institution of the OSC*. The OSC was started in August 2011. An unused clinic space was transformed into an operating room, with adjoining waiting, consultation, and postoperative recovery rooms (Figure 2). The original intention was to conduct five OSCs per week (replacing standard outpatient clinics). The average number was ultimately closer to three per week, operating on as intended a mean average of 6 patients per clinic (clinic duration is three hours).

After referrals were cherry-picked (choosing those most likely to require surgical intervention) by the OSCs respective consultant lead, patients were sent an appointment letter informing them about the possibility of immediate surgery. Patients could alter their appointment date (the NHS choose and book system) to decrease the probability of nonattendance [22].

The layout of the OSC is shown in Figure 2. After assessment by the consultant, if appropriate for surgery (criteria suitable for OSC surgery include skin lesions that once excised would require direct closure, small skin grafts, or simple local flaps; more complicated reconstructions requiring >30 minutes of operating time were not suitable; patients' anticoagulation status was considered on a case by case basis, with an instant skin prick INR test available for those on warfarin (INR < 2.5 preferred)), the patient was admitted and then taken either directly through to the operating room or for a short wait back to the waiting room whilst concurrent procedures finish. The consultant was on hand to assist the operating surgeon (senior house officer or registrar) with marking lesions and advice if required.

Postsurgery patients were taken into an adjoining postoperative space and given instructions by the nursing staff, whilst the next patient was prepped in the operating room. This arrangement minimised time delay. The clinics were staffed by one front desk administrative worker, one consultant, one operating surgeon (senior house officer or registrar), and two nursing staffs. Nursing staff retraining or reallocation (dependent on prior experience) was required to perform roles in the OSC such as circulating and assisting in the operative theatre. The grade and numbers of staff matched those of previous non-OSC clinics and thus, apart from the initial installation to equip the outpatient operating room, the OSC ran without a need to significantly alter resources or staffing.

## 2. Methods

The patient satisfaction questionnaire and OSC UK survey required local hospital board certification and the experimental methods observed the ethical principles of the Declaration of Helsinki.

### 2.1. OSC 18-Week Wait Performance and Measures of Departmental Capacity

With established data indicating the unit was struggling with 18 ww compliance, a further one-year analysis (May 2010 to April 2011) of departmental 18 ww performance was undertaken (18 ww referring to the interval between initial patient referral, usually from their family doctor to definitive care). The review commenced four months prior to and continued seven months after the OSCs institution. Additional associated measures of departmental capacity were also recorded, including the weekly totals of patients added to waiting lists.

### 2.2. Patient Satisfaction Survey

The patient satisfaction survey employed was adapted and validated by Javaid et al. when presenting their 2005 experience instituting a plastic surgery OSC (questions derived from the Newcastle Satisfaction with Nursing Scale) [2, 23]. A random sample of 200 patients was surveyed (from December 2010 to March 2011) at their two-month post-op follow-up visit, divided evenly between those receiving treatment in OSC and those treated in the conventional care pathway (100 OSC and 100 non-OSC conventional pathways). Patient consent was gained at the beginning of clinic, and the self-reported satisfaction survey completed at the end. The survey included four questions, using a 1–10 Likert scale (10 representing the highest degree of satisfaction), enquiring about patient's satisfaction with consultation, treatment in operation room, the quality/clarity of discharge instructions, and overall ratings of the service.

### 2.3. UK Survey of Plastic Surgery OSC Utilisation

A list of all adult plastic surgery units in the UK was obtained from the British Association of Plastic, Reconstructive and Aesthetic Surgeons website. A structured telephone interview was conducted with consultants, administration staff, or operational managers. The protocol involved an introductory email followed by two follow-up phone calls with a week's interval. Questions posed included whether the unit offered a “one-stop” or “see and treat” clinic facility. If yes, then what was that unit's experience of it and, if no, was there a reason why not?

## 3. Results

### 3.1. OSC 18-Week Wait Performance and Measures of Departmental Capacity

**Figure 3:**
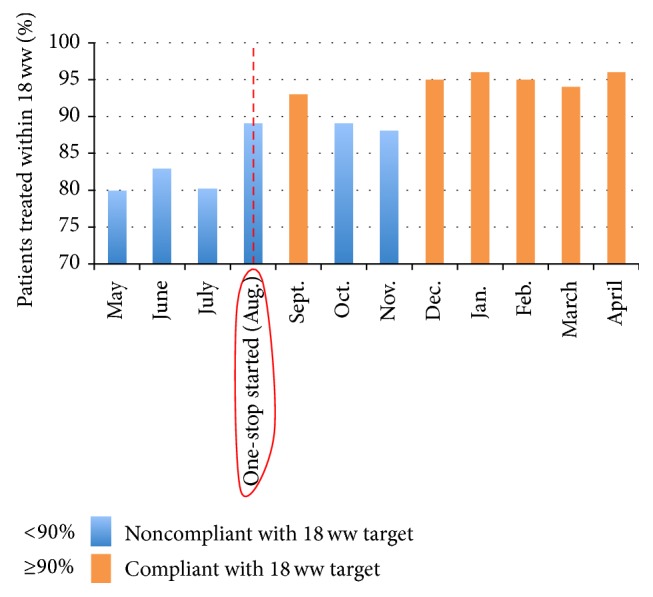
18-week wait compliance before and after institution of the one-stop clinic for minor skin cancer surgery, compliant months (>95%) in orange.

18 ww performance improved from a noncompliant pre-OSC mean average of 80% to a compliant mean average of 95% after OSC (compliance ≥ 90% Figure 3). Despite improvement, compliance was however narrowly missed in the months of October and November, when the clinic was first started (88 and 87%, resp.). This was due to improvements required in patient selection (i.e., selecting those more likely to require biopsy) and the fact that the number of OSCs operating per week increased over the first few months. Compliance was consistently achieved after this initial period.

**Figure 4:**
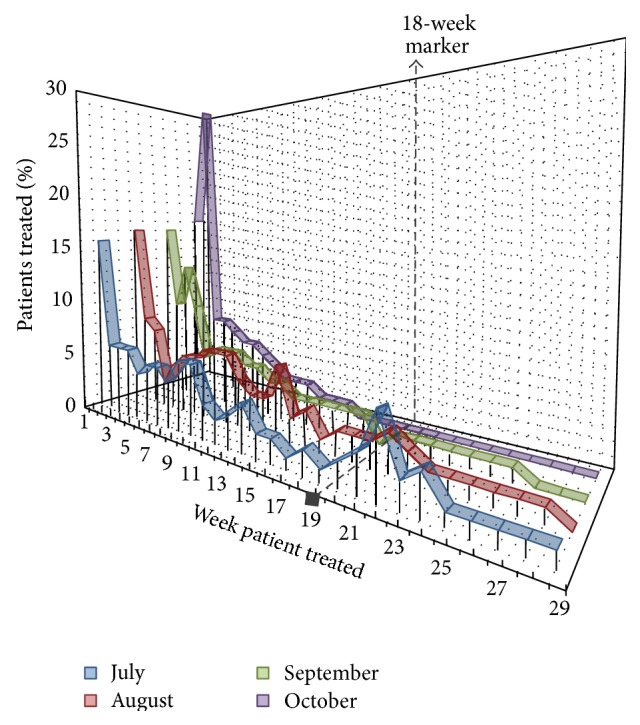
Four-month comparison of 18 ww performance, as a percentage distribution pattern of the week patients received treatment after initial referral. The 18 ww threshold is marked as a vertical black dotted line. The comparison commences one month prior to the OSCs institution in August. This figure relates to Figure 1, which provides exemplars to translate changing distributions.

The improvement in 18-week wait compliance was associated with improved measures of department capacity. This can be first illustrated by the changing distribution patterns, regarding which week patients received treatment after referral to the unit.  Figure 4 shows a four-month snap shot, illustrating a trend towards a leftward shift of the weekly distribution (after institution of the OSC) indicating that patients received care more consistently in the first few weeks of the 18 ww pathway, as opposed to later, or even after the 18-week threshold. The distribution changes from a wider bimodal (see Figure 1 for further explanation) distribution to the ideal negative ski slope distribution. Note that patients are treated at week “zero” or one, due to a prior backlog, the shortest realistic referral-treatment window being approximately two weeks.

**Figure 5:**
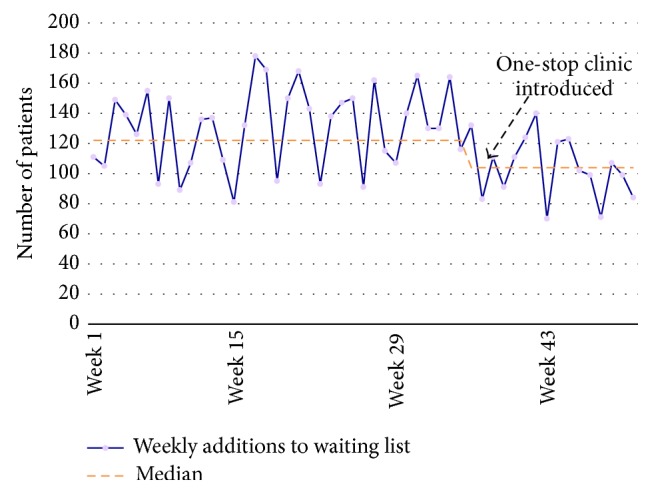
One-year review of 18 ww performance, showing the number of patients added to the departments' waiting list each week, the median trend represented by the orange dotted line, and weekly fluctuation in purple. The lower the number, the better the performance as patients are being treated in the OSC such that they are not added to the normal waiting list (i.e., standard non-OSC care pathway).

By measuring the number of patients added to departmental waiting lists each week, it was also possible to quantify the number of operating slots that were made available due to the OSC.  Figure 5 shows that after the OSC was introduced (~week 40), the median number of additions to the waiting list dropped by approximately 18 patients per week. This means that 18 patients per week were being treated in the OSC that would have normally been added to the standard care pathway waiting list. This translates to the department having an increased capacity of approximately 15% or 72 minor procedural operating slots per month.

### 3.2. Patient Satisfaction Survey Results

As patients completed the questionnaire in clinic, the response rate was 100%. 95% of the respondents expressed a high degree of satisfaction (score ≥7, scale 1–10) regarding their overall experience of the OSC, compared with 72% for the conventional pathway. A summary of the results comparing the OSC and conventional pathway is presented in Table 1.

### 3.3. UK Survey of Plastic Surgery Unit One-Stop Clinic Utilisation

**Figure 6:**
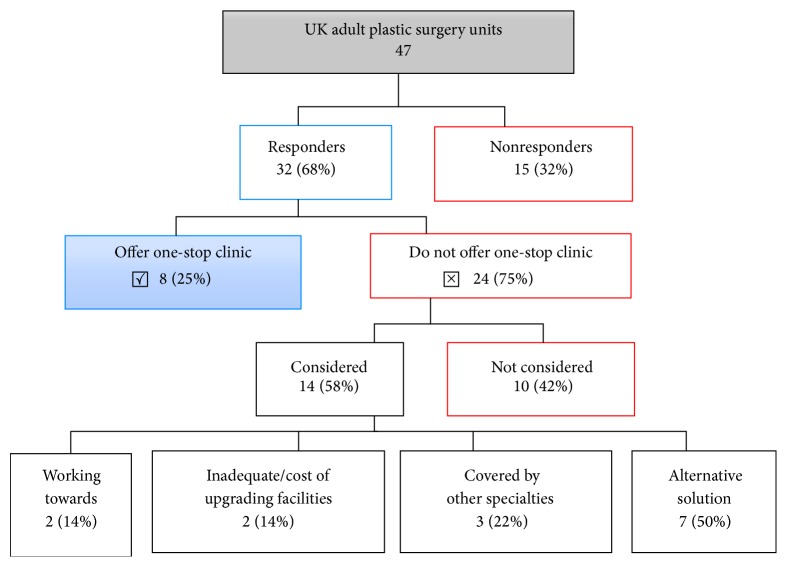
A survey of UK plastic surgery departments' utilisation of one-stop minor surgery skin cancer clinics.

The survey response rate was 68%, data obtained from 32 of the 47 units in the UK (Figure 6).

25% of UK units operate one-stop services for skin cancer; of those who did not, 42% offered no reason, 29% offered alternative services (such as outpatient local anaesthetic lists performed by nurse specialists or combined skin cancer clinics with dermatology), 13% associated the use of such clinics with other specialties, 8% stated they had inadequate facilities, and 8% stated they were developing a one-stop clinic model.

## 4. Discussion

With the advent of the OSC, waiting times in our department decreased and its 18 ww performance improved from a noncompliant pre-OSC mean average of 80% to a compliant mean average of 95% (target compliance 90%). The department's capacity increased by approximately 15%, equating to 72 minor procedural slots per month. With increased capacity, in addition to becoming target compliant, the unit was also able to respond to the 18 ww in the manner in which the target intends, such that the vast majority of patients were treated in the first few weeks of the pathway instead of later, at the end, or even beyond the 18 ww threshold.

Regarding the UK survey, we obtained responses from 32 (68%) of the 47 UK plastic surgery units. Reviews rationalising the adequacy of survey response rates have concluded that when an entire cohort of interest is surveyed (as opposed to a representative sample), a response rate of one-third is acceptable and two-thirds considered robust [24]. With a respondent rate exceeding two-thirds, we can extrapolate from our survey with confidence, accepting that a degree of responder bias cannot be ruled out (i.e., that respondents may have had a greater or lesser tendency, e.g., to employ OSCs in their units). As was guessed from the paucity of examples in the literature, only 25% of plastic surgery units offered an OSC service. Of the proportion that did not, almost half (42%) had not considered OSCs. This may be, as was our presurvey suspicion, due to a lack of “lean” type managerial thinking principles being employed within those units. Of those that had considered but still did not offer OSCs, half implemented “alternative solutions.” Most commonly, this was in the form of surgical nurse practitioner (SNP) led minor skin surgery lists. Otherwise, two units felt they had inadequate facilities, two were working towards an OSC, and three felt the service was already covered by another speciality. The reasons for offering SNP led lists included cost savings (SNP's salary versus a junior surgeon's) and that it maintained staffing continuity. There is little in the literature specific to plastic surgery, but both benefits have been shown with cardiac surgery SNPs [25]. The potential cost savings of SNPs are not well quantified and there have been no comparisons conducted between OSC and SNP led minor skin surgery services. Given that the units that ran SNP plastic surgery lists maintained the routine non-OSC pathway, requiring a minimum of two hospital visits, the other associated benefits of an OSC service are lost. A critique of nurse practitioner led service delivery is that it can lead to junior doctor's roles and learning opportunities being displaced [26]. Given that junior doctors felt that the OSC represents a particularly good training experience, both in our department* and* as cited by previous plastic surgery OSC studies [1, 2], this would be important to consider if SNPs were incorporated into the planning of a new OSC.

Our patient satisfaction questionnaire indicated that patients preferred the OSC compared with the non-OSC service (95% versus 72%). The levels of high satisfaction (≥7 out of 10) were similar to those reported by Javaid et al., in their 2005 plastic surgery OSC experience (93%), though their study did not include a comparison with their local non-OSC service. In our study, in keeping with feelings towards the overall service, patients reported higher satisfaction regarding their OSC consultation, treatment in the operating room, and discharge instructions. The reasons given (free text box questionnaire answers) were that the OSC was more convenient and caused less frustration, anxiety, and general life disruption. Similar feelings have been cited previously regarding OSC experiences in plastic surgery and other surgical specialities [2, 9, 11, 27]. It is likely that patients perceptions of the OSC were not only due to fewer hospital visits, but also as a result of the streamlined, simplified perioperative delivery of care when compared with the standard pathway. Successful examples of this in OSCs have been previously evidenced in an array of conditions (additional to the management of minor skin cancer surgery) treated by plastic surgery, including carpal tunnel syndrome, Dupuytren's contracture, and head and neck and breast cancer [10, 12, 13, 28–30]. In these examples, service adaptations included the use of portable nerve conduction studies for one-stop carpal tunnel clinics (testing carried out by the plastic surgeons as opposed to separate patient visits by neurophysiologists), procedures being carried out under local rather than general anaesthetic and modification to histopathology tests/protocols to reduce the diagnostic turnaround from weeks to hours.

Elements of patients' satisfaction with the OSC, such as patient consultations, were likely to have been influenced by factors not elicited or enquired about explicitly by the survey. For example, it is a reasonable inference that patients may have had more confidence in their OSC consultations due to them being exclusively undertaken by consultants, compared with conventional clinics, staffed by a mixture of junior and senior doctors. A general limitation of the questionnaire was that we did not explore such issues sufficiently and that it was conducted retrospectively, two months after patients perioperative experience. The interval between the OSC and survey completion may have increased the possibility of memory loss and confabulation. We feel that this was more likely to have occurred with reflections regarding clinic consultations and discharge instructions, as opposed to the overall evaluations of OSC/non-OSC services. An example of such bias would be a tendency to adjust ratings of, for example, their operating experience, to match the satisfaction level given to the overall service. This type of responder bias may be as much subconscious as conscious.

Other study design considerations involve our chosen outcome metrics and lack of formal cost analysis. The principle aim of the study was to demonstrate the OSC's efficacy using a national benchmark, the 18 ww, and map out OSC utilisation in UK plastic surgery. Additional outcome metrics that may have been studied include procedural complication and recurrence rates. Reviewing the evidence base, both factors have not been shown to increase significantly, and, employing “lean” thinking principles during our planning of the OSC, it was decided that such factors did not need to be explicitly investigated [1, 12, 13]. We did however take note of significant complications and across the study period no major complications were recorded. Partial local flap failure and post-op bleeds occurred, but not at a rate that significantly deviated from the non-OSC service. In terms of cost analysis, the department saved money by avoiding both fines (incurred by non-compliance to the 18 ww target) and the common prior practice of employing extra operative lists to address patient list backlogs. These were no longer required with the increased capacity afforded by the OSC. The supplementary capacity and operating slots were used by the department with cases that carried a routine NHS tariff. OSCs have already been shown to be cost effective with annual savings in one UK plastic surgery study being estimated to be around £150,000: £355 per OSC patient versus £449 for patients following the conventional pathway [2]. Using these figures to extrapolate for our unit, considering 72 cases per month following the OSC pathway, gives an annual cost saving of over £98,000, on top of which would be added the NHS tariff for the additional 72 cases that could be conducted on standard operating lists. It must be noted that these calculations are based on 2004 data not accounting for inflation so the absolute number value saved in the present day would be greater.

In comparing the OSC and non-OSC services, it should be acknowledged that the criteria applied to include cases appropriate for the OSC (see Section 1) meant that procedures undertaken were on average less complex and of shorter duration than those performed via the conventional pathway. With longer more complex cases, patients may again have felt more anxious and less satisfied with the non-OSC operative experience. However, when cases were matched, the junior surgeons reported that the OSC set-up resulted in a more economical process, such that procedures that would have taken 45–60 minutes were finished within 30 minutes. A more uniform and streamlined approach was achieved through various changes to practice. Information letters were sent to patients prior to clinic detailing the OSC rationale and possible outcomes of the visit. A consultant saw all referrals (as opposed to junior surgeons). Procedural consent and lesion marking was undertaken by the consultants prior to entering the operating room, obviating procedural elements that may delay a more junior surgeon. Nursing staffs' routine roles included the preparation of local anaesthetic, patient draping, pre- and postdebrief of the patient (including postoperative and follow-up instructions), and a general mandate to remove any obstacles to perioperative time. As opposed to in the main operating theatres, disposable instruments were used, trays' modified to contain only the instruments required. Patients skin was cleaned with alchohol (chlorhexidine) based solution, a sterile field maintained by using a single fenestrated (patient drapes with a predefined lesion window). Operating surgeons dressed in surgical scrubs and sterile gloves only. Template operative notes were devised, doubling up as a discharge letter.

There are of course many considerations that may be expanded upon in more detail with regard to setting up and running an OSC, such as training, staffing, procurement of equipment, and more detailed explanations of “lean” thinking philosophies. We have focused our discussion towards our primary outcome measures, the 18 ww, patient satisfaction, and the national survey of OSC plastic surgery utilisation. However, with regard to training, junior surgeons in our unit (as compared with surgeons coming to the end of their training) reported that the OSC was a good opportunity to operate on simple cases with the potential for consultant supervision as required. As conventional clinics were still being run, there was no impact noted on their ability to undertake outpatient consultations (given this was only performed by consultants in the OSC). OSC set-up and staff training costs could vary significantly, dependent on a department's infrastructure and ability to covert existing facilities. Using “lean” thinking and planning carefully, we were able to utilise preexisting hospital space and adapt available equipment. Examples included using a simple patient trolley as opposed to purchasing a new operative table, procuring disposable instrumentation obviating the need for autoclaves, and retraining staff to perform new/multiple roles (expanded upon in* “Organisation and institution of the OSC,*” Section 2). We have supplied estimates of the cost savings per patient in our department, earlier in the discussion, based upon previous cost analysis in the UK [2]. The overall aim of a OSC should be to improve department performance, in this case in terms of capacity, cost savings, and patient satisfaction, with essentially the same resources. To achieve this is likely to involve changes of practice based upon “Lean” type thinking principles, where care, is streamlined and existing staff often re-trained to perfor new and multiple roles. Procurement of facilities and equipment should be minimised.

## 5. Conclusion

OSCs are underutilised within UK plastic surgery, where a significant proportion of units acknowledge not having considered their use. This is despite associated improvements in waiting times, department capacity, and being preferred by patients who express high levels of satisfaction. We offer our considerations, planning, and positive local experience instituting an OSC service for minor skin cancer surgery. Our study indicates that the NHS in the UK is right to champion the use of see and treat delivery of care and encourage plastic surgery departments to consider their potential benefit.

## Figures and Tables

**Table 1:** Patients' views on the quality of service in one-stop clinics versus those treated within the standard care pathway^*∗*^.

Aspect of care	Patients who gave a score of 7 or higher	
	One-stop clinic	Standard pathway
Satisfaction with consultation	85%	70%
Treatment in operation room	82%	72%
Quality and clarity of discharge instructions	80%	64%
Overall rating of quality of service	95%	72%

^*∗*^10 point likert scale (10 best) self-reported survey.
